# Composition and Diversity Characteristics of Gut Microbiota during the Development of *Telchinia issoria* (Lepidoptera: Nymphalidae)

**DOI:** 10.1002/ece3.73596

**Published:** 2026-04-30

**Authors:** Xin Yang, Liu liu Dong, Xiao xiao Jin, Xu jie Liu, Min Gao, Jie Fang

**Affiliations:** ^1^ School of Life Sciences and Medical Engineering Anhui University Hefei China

**Keywords:** gut microbiome development, larva, lepidoptera, life stage, *Telchinia issoria*

## Abstract

Ramie ( *Boehmeria nivea* ) was a traditional economic crop of high commercial value, whose cultivation was threatened by the leaf‐feeding pest *Telchinia issoria*. This study investigated how the gut microbiota of *T. issoria* shifted across its larval, pupal, and adult stages using 16S rRNA amplicon sequencing. We found that Pseudomonadota and Bacillota dominated across all stages, with stage‐specific enrichments of key genera: *Burkholderia‐Caballeronia‐Paraburkholderia* in early larvae, *Acinetobacter* and *Culicoidibacter* in mid‐instars, *Serratia* in late larvae, *Enterococcus* in pupae, and *Pseudomonas* in adults. Alpha diversity exhibited a U‐shaped pattern during larval development, decreasing initially before rising again, with the lowest overall diversity observed in the pupal stage. Beta diversity confirmed distinct community structures in pupae and adults. Functionally, as predicted by PICRUSt2 based on 16S rRNA gene sequencing data, carbohydrate metabolism was enriched in pupae, whereas pathways associated with amino acid, cofactor, and vitamin metabolism were significantly decreased relative to other developmental stages. Correlation analysis suggested that elevated temperature may contribute to the decreased diversity observed in this study, which warranted further verification under controlled temperature gradients. This work establishes a foundational understanding of stage‐specific microbial symbiosis in *T. issoria* and offers insights for future research into lepidopteran gut microbial ecology and potential biocontrol applications.

## Graphical abstract

Ramie ( *Boehmeria nivea* ) is a traditional economic crop of high commercial value, whose cultivation is threatened by the leaf‐feeding pest *Telchinia issoria*. This study investigated how the gut microbiota of *T. issoria* shifts across its larval, pupal, and adult stages using 16S rRNA amplicon sequencing.

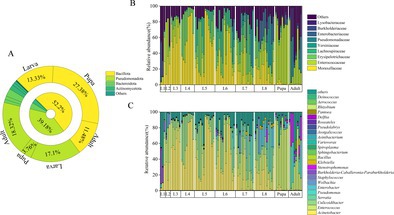

**Abbreviations**
L1
: First‐instar larvae
L2
: Second‐instar larvae
L3
: Third‐instar larvae
L4
: Fourth‐instar larvae
L5
: Fifth‐instar larvae
L6
: Sixth‐instar larvae
L7
: Seventh‐instar larvae
L8
: Eighth‐instar larvae
LDA
: The linear discriminant analysis
LEfSe
: The linear discriminant analysis effect size
NMDS
: Nonmetric multidimensional scaling
OTU
: Operational Taxonomic Units
*PICRUSt*_*2*_
: Phylogenetic investigation of communities by reconstruction of unobserved states 2

## 1 Background

The insect gut is a tubular organ comprising the foregut, midgut, and hindgut. Digestion and absorption occur predominantly in the midgut, whereas the hindgut serves as the principal site for microbial colonization (Zhang, Zhang, and Lu 2022). Over millions of years of continuous evolution, insects and their gut microbiota have developed a coordinated symbiotic relationship, enabling the host to adapt to diverse ecological niches (Wang et al. 2024; Dillon and Dillon 2004). These resident microorganisms play indispensable roles in host growth and development, contributing to functions such as immune defense, detoxification, nutrient provisioning, digestion, and absorption (Zhang, Feng, et al. 2022; Duplais et al. 2021; Xia et al. 2023; Zheng et al. 2019; Luo et al. 2019; Jing et al. 2020; Cheng et al. 2017). The functional capacity of the gut microbiota depends on its specific composition, which includes both beneficial symbionts and potential pathogens (Dillon and Dillon 2004). For instance, Vibrio species may facilitate the growth of other gut microbes and aid in protecting the host from toxins (Satchell 2015). Conversely, *Bacillus thuringiensis* can induce cell membrane perforation and cell death in insects like the boll weevil ( *Anthonomus grandis* ) (Ribeiro et al. 2024). Key factors shaping this diversity include host‐specific characteristics (e.g., species and maternal transmission) and external environmental factors (e.g., diet and habitat) (Hasan and Yang 2019; Xie et al. 2024; Shan et al. 2024; Lü et al. 2019). As poikilotherms, insects are highly susceptible to temperature fluctuations and extreme climatic events. Studies have demonstrated that temperature shifts can substantially alter the composition and functional profile of the gut microbiota in diverse insect species, including *Reticulitermes flavipes* , *Zygogramma bicolorata*, and *Bactrocera dorsalis* (Arango et al. 2021; Singh et al. 2025; Ayyasamy et al. 2021). Host development, marked by substantial morphological and physiological changes, is another major driver of microbial community dynamics. The gut microbiota undergoes continuous restructuring throughout this process (Lü et al. 2023; Ayayee et al. 2022). Drawing an analogy from human studies, the infant gut microbiota rapidly increases in diversity with age, reaching a stable, adult‐like state by approximately 3 years of age (Panda et al. 2014). In advanced age, the number of beneficial bacterial species, such as *Bifidobacterium* and *Lactobacillus*, in the gut microbiota decreases significantly (Kumar et al. 2016). Insect development via metamorphosis creates distinct ecological niches for larval and adult stages, thereby reducing intraspecific competition (Rolff et al. 2019). This life‐history strategy enables the host to maintain beneficial symbionts across life stages while simultaneously segregating and restructuring microbial communities between larval and adult forms (Kowallik and Mikheyev 2021). Consistent with this dynamic change, the composition of gut microbial communities exhibits significant, stage‐specific variation during the successive developmental stages of insects, as documented in species including *Apis dorsata* , *Zeugodacus tau*, and *Periplaneta americana* (Chen et al. 2023; Noman et al. 2021; Saraithong et al. 2017).

Lepidoptera (butterflies and moths), comprising approximately 160,000 described species, represents the second‐largest insect order, many species of which are significant agricultural pests causing substantial crop losses. The lepidopteran gut presents a hostile environment for microbial colonization. This is characterized by general hypoxic conditions and, in larvae, an intensely alkaline midgut with pH values ranging from 7 to 12 (Rousk et al. 2009; Johnson and Barbehenn 2000). Furthermore, holometabolous development (complete metamorphosis through egg, larval, pupal, and adult stages) poses an additional challenge for persistent bacterial colonization (Wang et al. 2020). Each developmental transition involves molting and extensive remodeling of the gut epithelium (Romanelli et al. 2016). Despite these harsh conditions, numerous studies confirm that resident bacteria influence basic physiological functions in Lepidoptera (Xia et al. 2017). The gut microbiota itself undergoes dynamic changes aligned with host development (Chen et al. 2018). During early stages, microbial communities experience ecological succession, involving bacterial colonization, cooperative interactions, and competition for spatial niches (Sanaei et al. 2024; Figueiredo and Kramer 2020). These dynamics culminate in the establishment of a more stable adult microbial community, which often differs markedly from that of the larval stage (Li et al. 2023; Wang et al. 2023). For instance, in *Brithys crini*, while Proteobacteria dominate both larval and adult guts, the predominant genera shift from *Empedobacter* in larvae to *Serratia* in adults (González‐Serrano et al. 2020). Understanding lepidopteran gut microbiota offers promising applications. In pest management, such research is paving the way for strategies that reduce reliance on chemical pesticides, thereby minimizing adverse impacts on human health and the environment (Zhang et al. 2023). The larval stage is a crucial period for nutrient accumulation and rapid growth‐ along with its unique microbiota, opens avenues for bioremediation (Xu et al. 2025). Recent research indicates that lepidopteran larvae, through the combined action of endogenous enzymes and gut microorganisms, can metabolize materials such as polyethylene (Tang, Chen, et al. 2025).

The larvae of *Telchinia issoria* (Lepidoptera: Nymphalidae) are a significant pest of the economically important crop ramie ( *Boehmeria nivea* , Rosales: Urticaceae), causing substantial damage particularly during the larval feeding stage (Espeland et al. 2018). This species undergoes eight larval instars, completes at least two generations annually, and exhibits generational overlap influenced by environmental variables including temperature, humidity, and rainfall (Hellmann 2002). Current research on insect gut microbiota is largely centered on established popular insects such as *Ceratitis capitata* and *Aedes aegypti* , with analyses frequently restricted to a single developmental time point (Bel Mokhtar et al. 2022; Harrison et al. 2023). Consequently, longitudinal studies that track microbial community dynamics across an insect's life cycle, along with comparative analyses of compositional and diversity shifts between consecutive developmental stages, remain notably limited. Furthermore, although environmental factors are recognized as major drivers of insect biology, their specific effects on the gut microbiota of *T. issoria* are poorly understood. To address these knowledge gaps, we focused this study on determining how host development and key environmental factors collectively shape the gut microbiome of *T. issoria*. We characterized the gut bacterial communities across larval, pupal, and adult stages via high‐throughput 16S rRNA gene amplicon sequencing and evaluated their correlations with three pertinent environmental parameters. Elucidating these relationships is critical for advancing targeted biological control strategies against this agricultural pest (Lamichhane et al. 2017). This work establishes a foundational framework for future comparative studies on microbial symbiosis within Lepidoptera and enhances our broader ecological understanding of host–microbe interactions, thereby contributing to the development of more sustainable pest management approaches.

## 2 Materials and Methods

### 2.1 Sample Collection and Gut Dissections

In this study, eggs of *Telchinia issoria* were collected from Huangshan Scenic Area, Huangshan City, Anhui Province, China (30.07117235° N, 118.16788854° E), at an elevation of 310–320 m, in village and farmland habitats. The eggs were brought back to the laboratory. Once Eggs hatched, the larvae were continuously fed with fresh *B. nivea* leaves. The newly emerged adults were fed with a 10% honey solution for nutrition.

We collected larvae, pupae, and adults in good growth condition that had been in their respective developmental stages for 3 days. Prior to dissection, samples were subjected to starvation treatment, euthanized with CO_2_, completely soaked in 75% ethanol for 3 min, and then rinsed three times with sterile water. For the larvae, early smaller‐sized larvae were dissected under a microscope using sterile instruments to obtain intestinal samples. For the adults, we cut off the wings on the thorax and then performed surface sterilization and removed the intestines. For the pupae, an incision was made at the posterior end using tweezers to expose and remove the intestinal tract, which was then placed into a centrifuge tube. The larvae, pupae, and adults were dissected with sterile instruments and immediately frozen. All samples were frozen at −80°C until DNA extraction. The whole process of gut samples was carried out under sterile conditions. Ultimately, we obtained 83 samples, 67 samples for larvae (3 samples for 1st and 2nd instars, 6 samples for 3rd instar, 8 samples for 4th instar, 12 samples for 5th, 6th, and 8th instars, and 11 samples for 7th instar), 8 samples for pupae and adults. All samples were regarded as biological replicates rather than technical replicates. According to instar characteristics, larvae were further divided into three groups: early larval stage (1st –2nd instars), middle larval stage (3rd –6th instars), and late larval stage (7th –8th instars).

### 2.2 Measurements of Morphological Index and Environmental Factor

Using vernier calipers with an electronic analytical balance measured the body length, body weight and head capsule width of the larvae which required for the experiment. Using an environmental monitor to monitor the temperature(Room temperature: 25°C ± 5°C), relative humidity(70% ± 5%) and light intensity of the environment every 10 min. The monitor was placed 10 cm directly above the breeding box to ensure the accuracy of the monitoring data. The average environmental data of the duration of survival of the larvae was used as the environmental data.

### 2.3 DNA Extraction, PCR Amplification, and Sequencing

DNA was extracted using E.Z.N.A. DNA Kit (Omega Bio‐tek, Norcross, GA, U.S.). Subsequently, the Extractions was used to PCR amplification, amplicon library preparation, and high‐throughput 16S rRNA sequencing. The V4‐V5 region of the bacteria 16S ribosomal RNA gene were amplified using Universal primers 515F (5′‐GTGCCAGCMGCCGCGG‐3′) and 907R (5′‐CCGTCAATTCMTTTRAGTTT‐3′). The PCR were 95°C for 2 min, followed by 25 cycles of 95°C for 30 s, 55°C for 30 s, and 72°C for 30 s, with a final extension at 72°C for 5 min. PCR reactions were performed in triplicate 20 μL mixture containing 4 μL of 5 × FastPfu Buffer, 2 μL of 2.5 mM dNTPs, 0.8 μL of each primer (5 μM), 0.4 μL of FastPfu Polymerase, and 10 ng of template DNA.

AxyPrep DNA Gel Extraction Kit (Axygen Biosciences, Union City, CA, U.S.) and Quantus Fluorometer were used for purification and quantification, respectively. Subsequently, Sequencing was conducted on the Illumina MiSeq platform (PE300; Shanghai Oumeng Biotech Co. Ltd., Shanghai, China).

The raw sequences results used the fastp software (version 0.20.0) controlled quality, and used the FLASH software (version 1.2.7) carrying out double‐ended sequence stitching (Bolger et al. 2014; Magoč and Salzberg 2011). the UPARSE software (version 7.1) was used to cluster clean reads into Operational Taxonomic Units (OTU) based on a 97% similarity threshold (Edgar 2013). The RDP classifier (version 2.2) was used to annotate the taxonomic classification of each sequence by combining with the Silva 16S rRNA database (v138) with a confidence threshold of 80% (Quast et al. 2013).

### 2.4 Bioinformatics Analysis

Rarefaction was performed using Mothur (v.1.21.1) to normalize sequencing depths across samples, generating a rarefied OTU table. On the basis of this dataset, alpha diversity indices, including Simpson indices and Shannon indices, were calculated (Schloss et al. 2009). Statistical analysis of microbial Alpha diversity used the nonparametric Kruskal–Wallis (KW) test to assess significant differences. Microbial Beta diversity was analyzed based on Bray–Curtis distances to compare the similarities in microbial community structure among samples. Nonmetric multidimensional scaling (NMDS) plots were constructed using Bray–Curtis. Adonis were performed using R vegan package to assess the statistically significantly difference of bacterial communities' composition between samples. Linear Discriminant Analysis Effect Size (LEfSe) was used to identify dominant populations among samples (Segata et al. 2011). Using PICRUSt_2_(http://picrust.github.io/picrust/tutorials/genome_prediction.html) to comparison the 16S rRNA V4‐V5 region sequencing results with the KEGG databases to infer the functional alteration of microbiota in different samples (Langille et al. 2013). The OTU data obtained were used to generate BIOM files formatted as input for PICRUSt_2_ with the make.biom script usable in the Mothur.

### 2.5 Statistical Analysis

Statistical analysis was performed using SPSS 22.0 software package and GraphPad Prism 9. Correlation analysis between gut microbiota and morphological index as well as environmental factors was conducted using the correlation analysis of *spearman*. Performing multiple test corrections used FDR correction (Benjamini‐Hochberg). (**: FDR < 0.001, *: FDR < 0.05).

## 3 Results

### 3.1 Composition of Gut Bacteria Throughout the Life Cycle of *Telchinia issoria*

After quality filtering and preprocessing, archaeal, mitochondrial, and chloroplast sequences were filtered out. A total of 2,953,203 effective tags were obtained from 83 samples, with an average of approximately 35,000 effective sequences per sample and a mean sequence length of 419 bp. Shannon–Wiener curves, Species accumulation curves reflected the species abundance and saturated sampling depth of the samples, indicated that the sequencing data samples were sufficient (Figure S1A,B). Good's coverage was used to show the completeness of sequencing, and the coverage of each sample was higher than 99%, indicated that this study obtained the detection of most valid species in the sample (Table S1).

**FIGURE 1:**
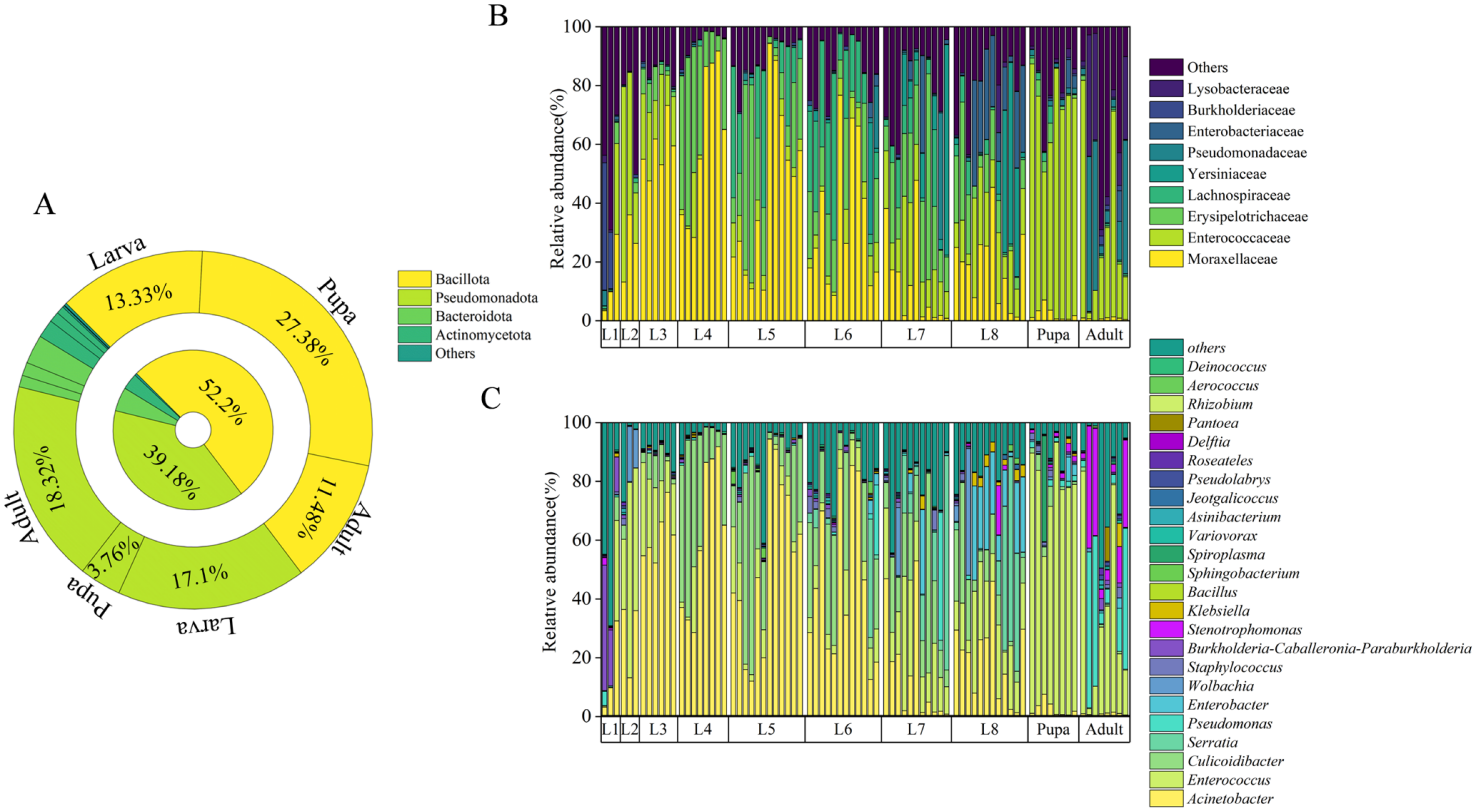
Microbial community composition across developmental stages of *T. issoria*. (A) Top 5 in relative abundance of microorganism composition at the phylum level. (B) Top 10 in relative abundance of microorganism composition at the family level. (C) Heat map of the top 25 genera in terms of relative abundance at the genus level.

The taxonomic analysis at phylum level showed that the dominant phyla in the development stage of *T. issoria* were Pseudomonadota (formerly Proteobacteria), Bacillota (formerly Firmicutes), followed by Actinobacteria and Bacteroidete. Bacillota had the highest relative abundance in pupal stage; instead, Proteobacteria had higher relative abundance in larval and adult stage (Figure 1A). At the family level, Moraxellaceae and Erysipelotrichaceae dominated the gut microbiota during the larval stage; Enterococcaceae was the dominated family in pupal stage, while Pseudomonadaceae was common in adult stage (Figure 1B). At the genus level, *Acinetobacter* was abundant during larval stage, but was relatively rare in pupae and adults. *Burkholderia‐Caballeronia‐Paraburkholderia* was common in the 1st‐instar larvae and *Culicoidibacter* became the main genus in the 4th, 5th, and 6th‐instar larvae. *Klebsiella* and *Serratia* were enriched in 8th instar larvae. The pupae were primarily colonized by *Enterococcus*, while the adults were dominated by *Pseudomonas* and *Stenotrophomonas* (Figure 1C).

### 3.2 Changes in Microbial Community Diversity Across the *Telchinia issoria*

**FIGURE 2:**
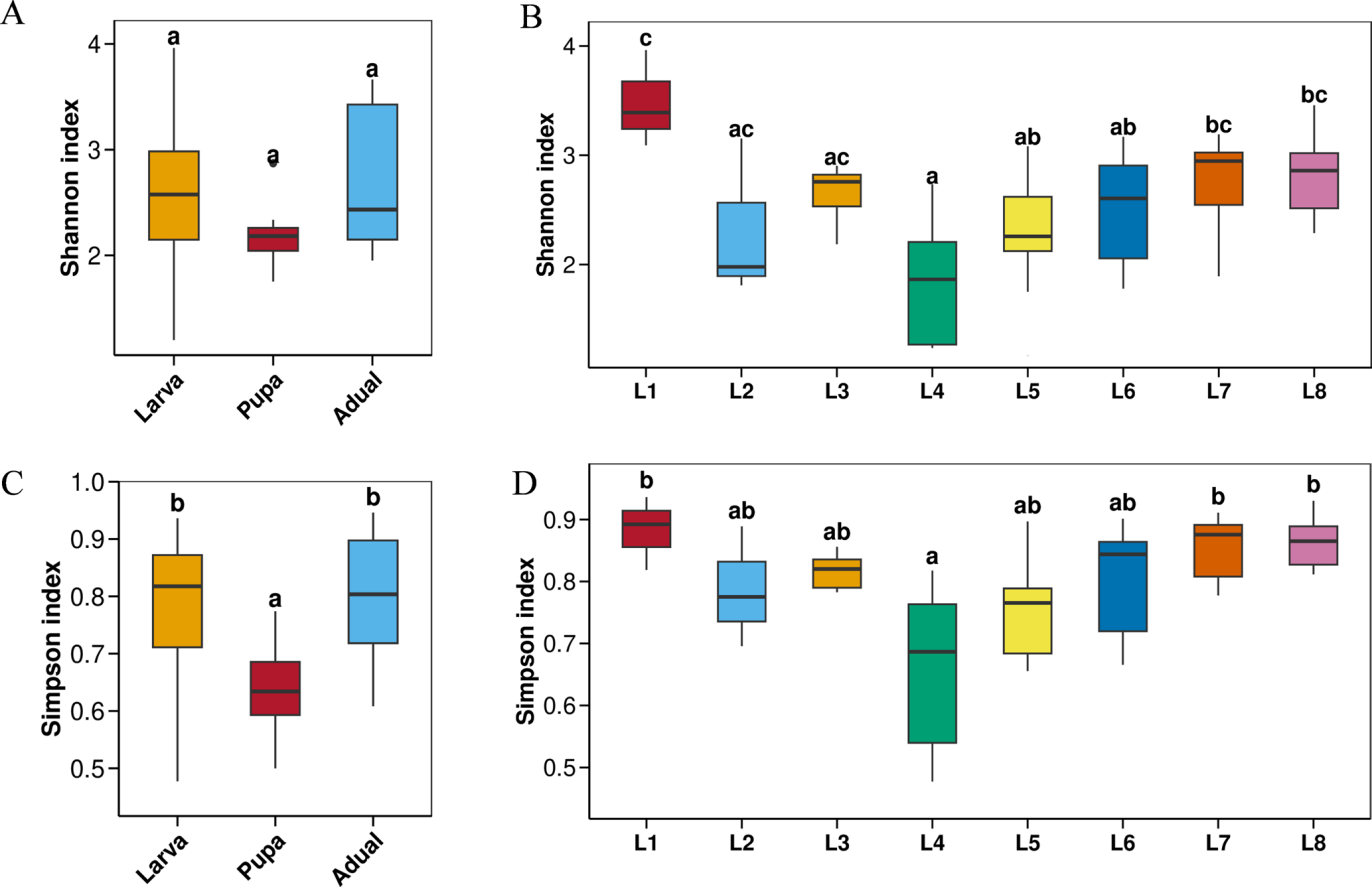
Distribution of alpha diversity of gut‐associated microbiota across developmental stages of *T.issoria*. (A) Shannon index at the larval, pupa. and adult stage. (B) Shannon index at the different larval stage. (C) Simpson index at the larval, pupa. and adult stage. (D) Simpson index at the different larval stage. Different lowercase letters denote significant differences between different life stages (*p* < 0.05, Kruskal–Wallis test).

The alpha diversity indices fluctuated throughout the life stages (Figure 2A–D and Table S1). During the process of transformation from larvae to adults, the diversity of gut microbiota initially decreased and then increased. The diversity of 1st‐instar larvae was highest; it decreased from the 2nd to 4th instars and generally increased from the 5th to 8th instars. The intergroup difference test of the alpha diversity index revealed the Simpson diversity index at the pupal stage was significantly lower than that at the larval stage and adult stage (*p* < 0.05, Kruskal–Wallis test). The microbiota diversity was significantly lower in the 4th‐instar larvae than in the 1st‐instar larvae and 7th, 8th‐instar larvae (*p* < 0.05). (Table S2).

**FIGURE 3:**
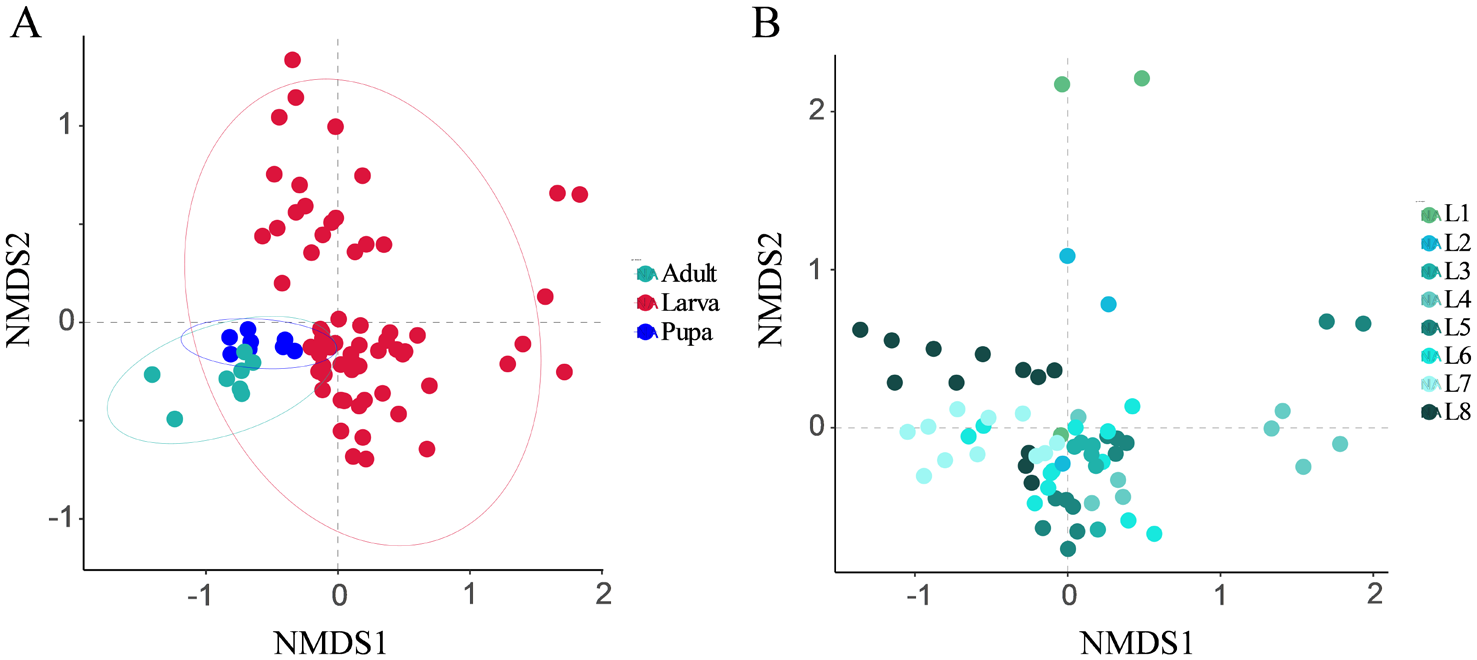
Distribution of beta diversity of gut‐associated microbiota across developmental stages of *T. issoria*. Two‐dimensional NMDS of microbial communities used the Bray–Curtis distance calculation to measure (A) larval, pupal and adult stages and (B) different larval stage samples.

On the basis of the Bray–Curtis method, the pupae and adults possessed their own distinguishable communities, albeit with slight overlap with that of other developmental stages (Figure 3A) (NMDS, stress = 0.144 PERMANOVA: F = 13.339 *p* < 0.001 permutations = 999). The microbial structures during the entire larval period showed overlapping similarities, but there also were differences. (Figure 3B) (NMDS, Stress = 0.144 PERMANOVA: F = 5.166 *p* < 0.001 permutations = 999). According to Adonis pairwise comparison, the bacterial communities of early and late larvae were different. Notably, adjacent larval instars tended to cluster together, suggesting gradual, stage‐by‐stage succession in community structure rather than abrupt shifts (Table S3). This indicated that the gut microbiota community structure in *T. issoria* differed across developmental stages.

### 3.3 Significantly Different Bacterial Communities Across the Development of *T. issoria*

**FIGURE 4:**
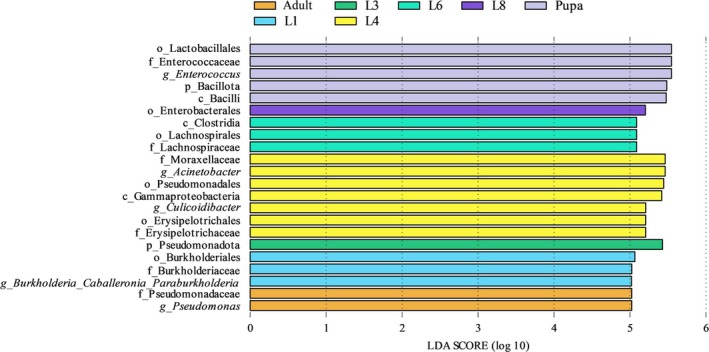
LEfSe analysis showing significant differences of microbiota species from phylum to genus. LEfSe Bar diagram for different life stages with LDA scores higher than 5.0. A higher LDA score represents that this microbiota taxon has a greater contribution to the differences.

LEfSe analysis (Linear Discriminant Analysis Effect Size) was performed to identify taxa that were significantly enriched across the developmental stages of *T. issoria*, from the phylum to the genus level (Figure 4, Figure S2). Several stage‐specific biomarker taxa were identified. During the larval stages: the 1st instar showed enrichment of three taxa spanning multiple taxonomic ranks (1 order, 1 family, and 1 genus); the 3rd instar was enriched for one phylum‐level taxon; the 4th instar was characterized by seven enriched taxa (1 class, 2 orders, 2 families, and 2 genera); the 6th instar had three enriched taxa (1 class, 1 order, and 1 family); and the 8th instar was enriched for one taxon. Beyond the larval phase, the pupal and adult stages were enriched with five and two taxa, respectively. At the genus level, *Burkholderia‐Caballeronia‐Paraburkholderia* was significantly enriched in 1st‐instar larvae; *Acinetobacter* and *Culicoidibacter* were highly enriched in 4th‐instar larvae; *Enterococcus* was abundant in pupae, whereas *Pseudomonas* was abundant in adults.

**FIGURE 5:**
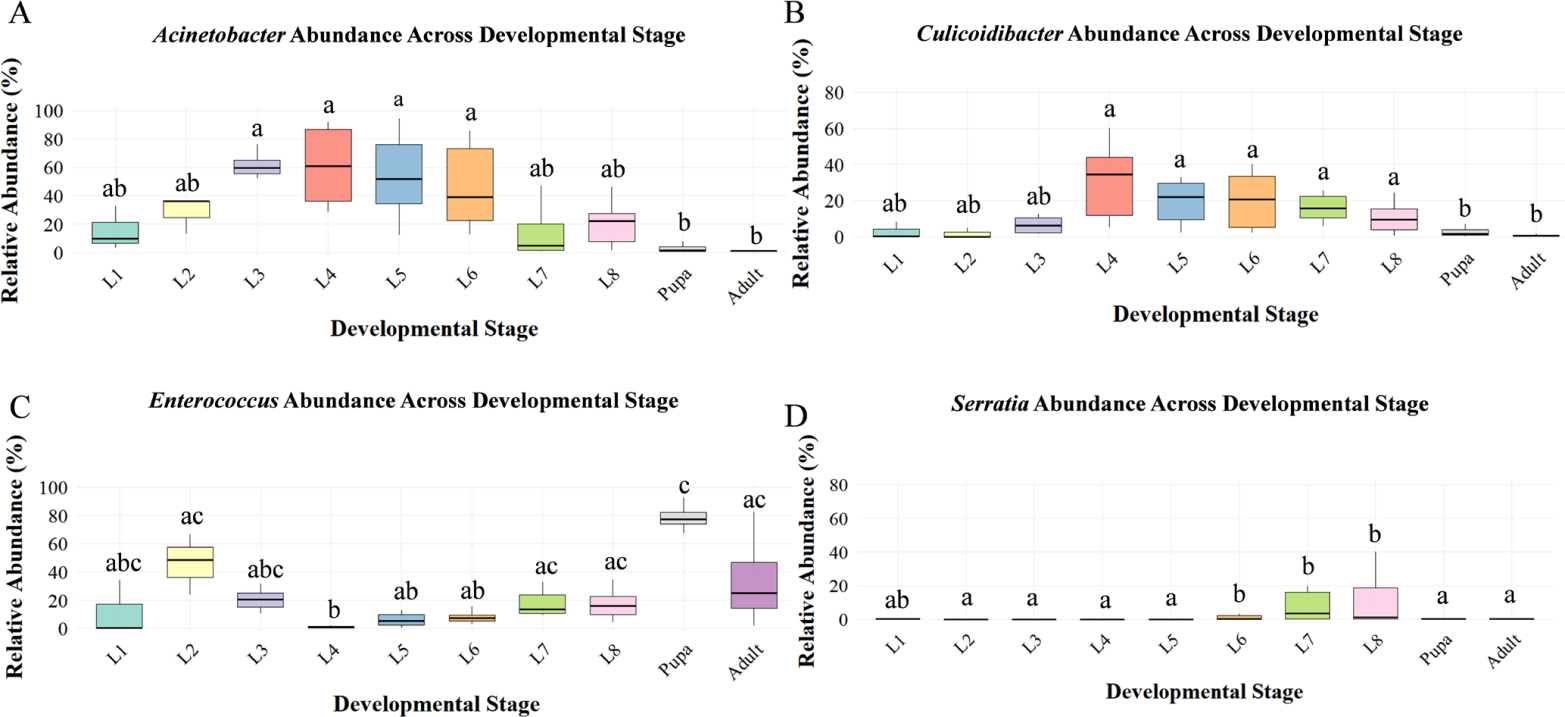
(A) Significance test of genus differences in the gut microbiota of *T. issoria* larvae. *p* ≤ 0.05.

Further analysis of the core gut microbiota of *T. issoria* revealed that the abundance of gut microbiota dynamic changes with host growth. The relative abundance of *Acinetobacter* and *Culicoidibacter* gradually increased from the 1st to the 4th instar, peaked in the 4th instar, then the overall trend is downward from the 5th to 7th instar. Their abundances reached the lowest levels during the pupal and adult stages, which were significantly lower than those observed during mid‐larval development. In contrast, *Enterococcus* exhibited a more complex pattern: its abundance gradually increased from the 1st to the 2nd instar, decreased from the 3rd to the 4th instar, rose again from the 5th instar through the pupal stage, and subsequently declined in adults. The abundance of *Serratia* showed significantly higher relative abundance in the final larval stage compared to all other developmental stages. (Figure 5).

### 3.4 Functional Prediction of Microbiota in the Development of *T. issoria*

**FIGURE 6:**
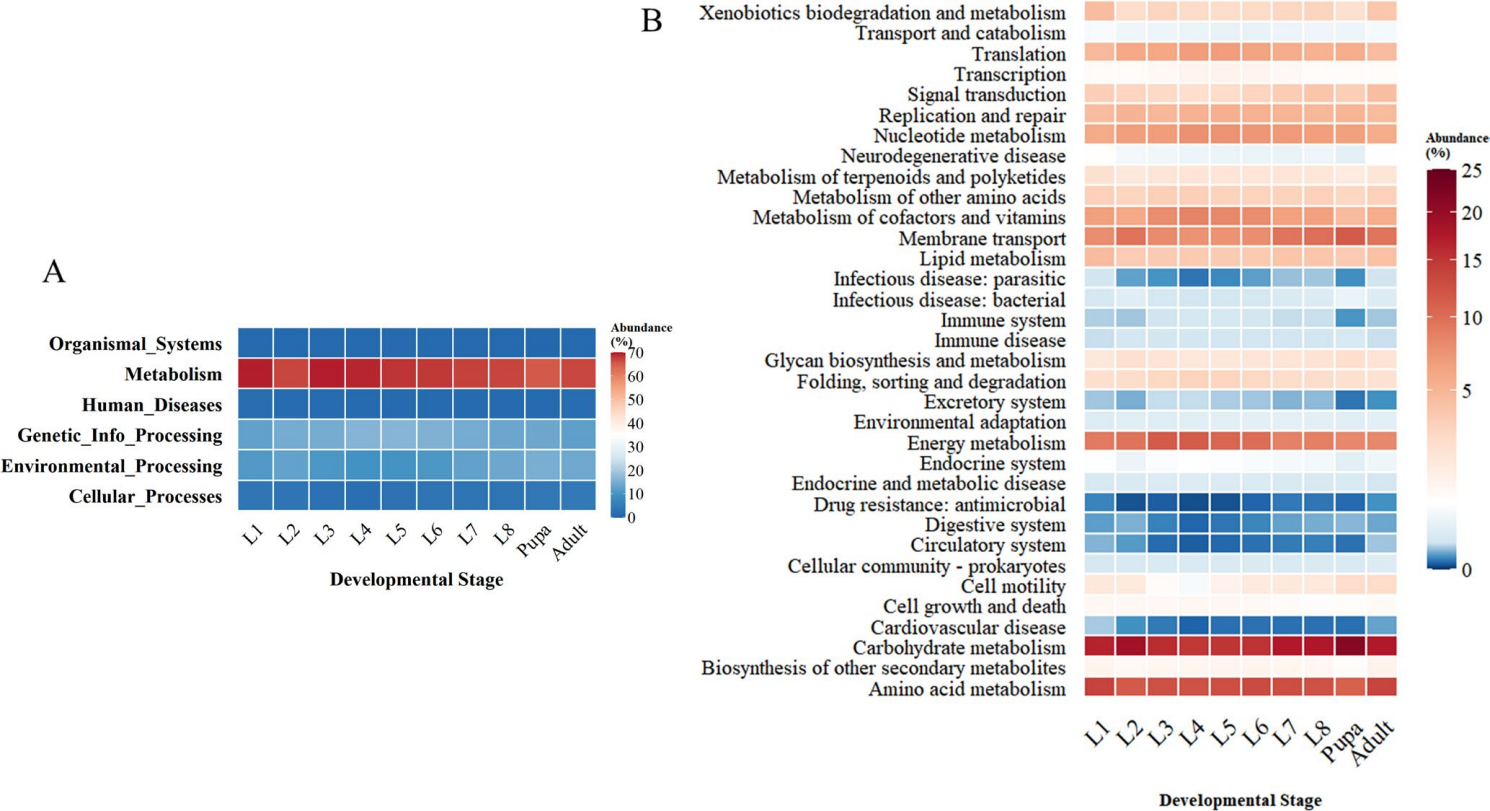
Inferred functions of microbiota community associated of *T. issoria*. Annotations of the KEGG pathways for gut microbiota gene functions. (A) KEGG primary pathway annotation level. (B) KEGG secondary pathway annotation level.

The relative abundances of KEGG pathways were predicted by PICRUSt_2_ based on 16S rRNA gene sequences. Metabolism, Genetic Information Processing, Environmental Information Processing, Human Diseases, Cellular Processes, and Organismal Systems (Table S4). Among them, metabolism accounts for the highest proportion, followed by Genetic Information Processing and Environmental Information Processing (Figure 6A). The key secondary metabolic functions included amino acid metabolism, carbohydrate metabolism, metabolism of cofactors and vitamins, energy metabolism, among others (Figure 6B).

**FIGURE 7:**
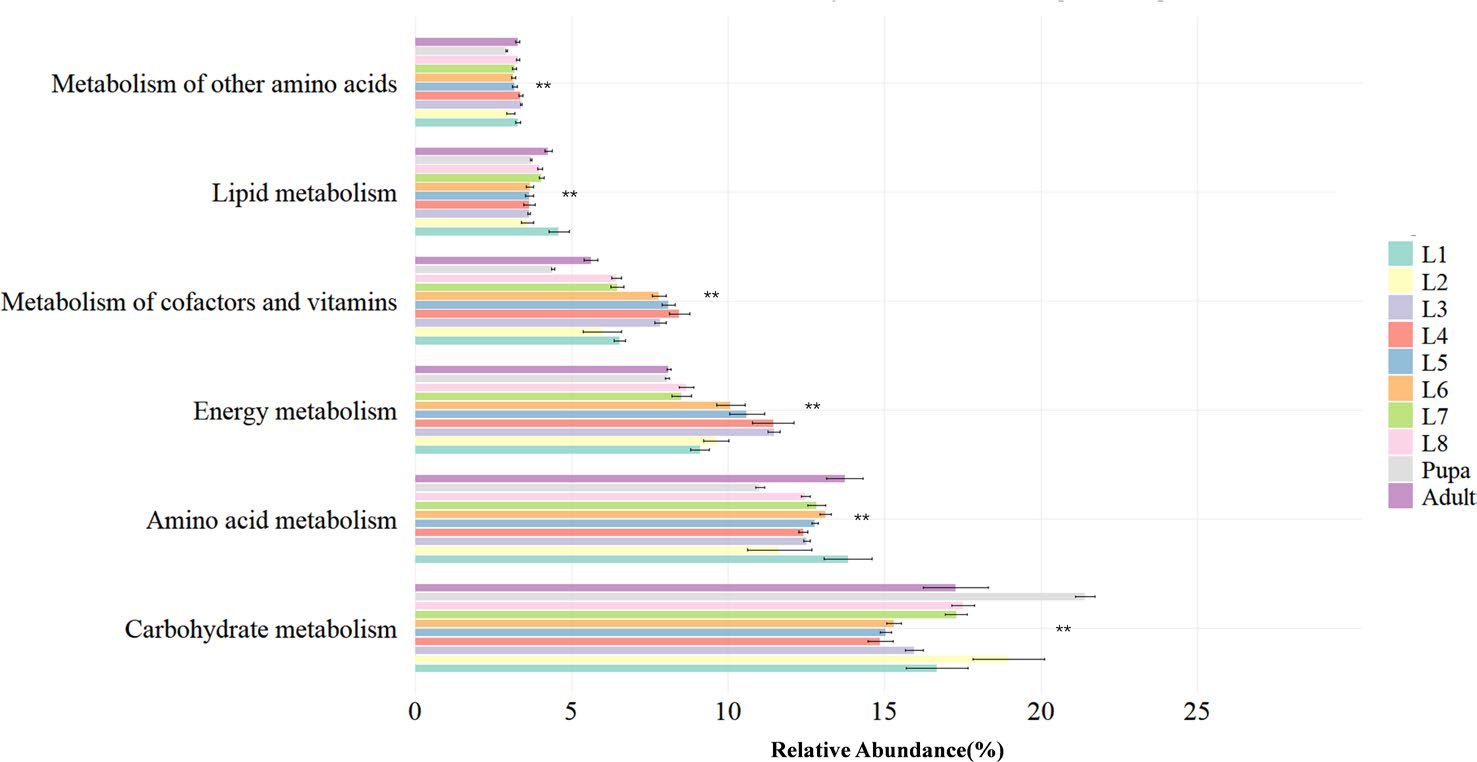
Analysis of gene functional differences in the gut microbiota of *T. issoria*. *: *p* ≤ 0.05, **: *p* ≤ 0.01.

Further analysis of the functional differences of microbiota genes indicated that the abundance of amino acid metabolism and lipid metabolism functions was the highest stage1st‐ instar larvae and adults. Additionally, the functions related to energy metabolism and metabolism of cofactors and vitamins were more abundant in the 4th‐instar larvae. The functional richness of carbohydrate metabolism was higher in the gut of pupae. Functions of metabolism of other amino acids, amino acid metabolism, and metabolism of cofactors and vitamins in pupae were significantly lower than other stages (Figure 7).

### 3.5 Correlation between Gut Microbiota and Various Factors

**FIGURE 8:**
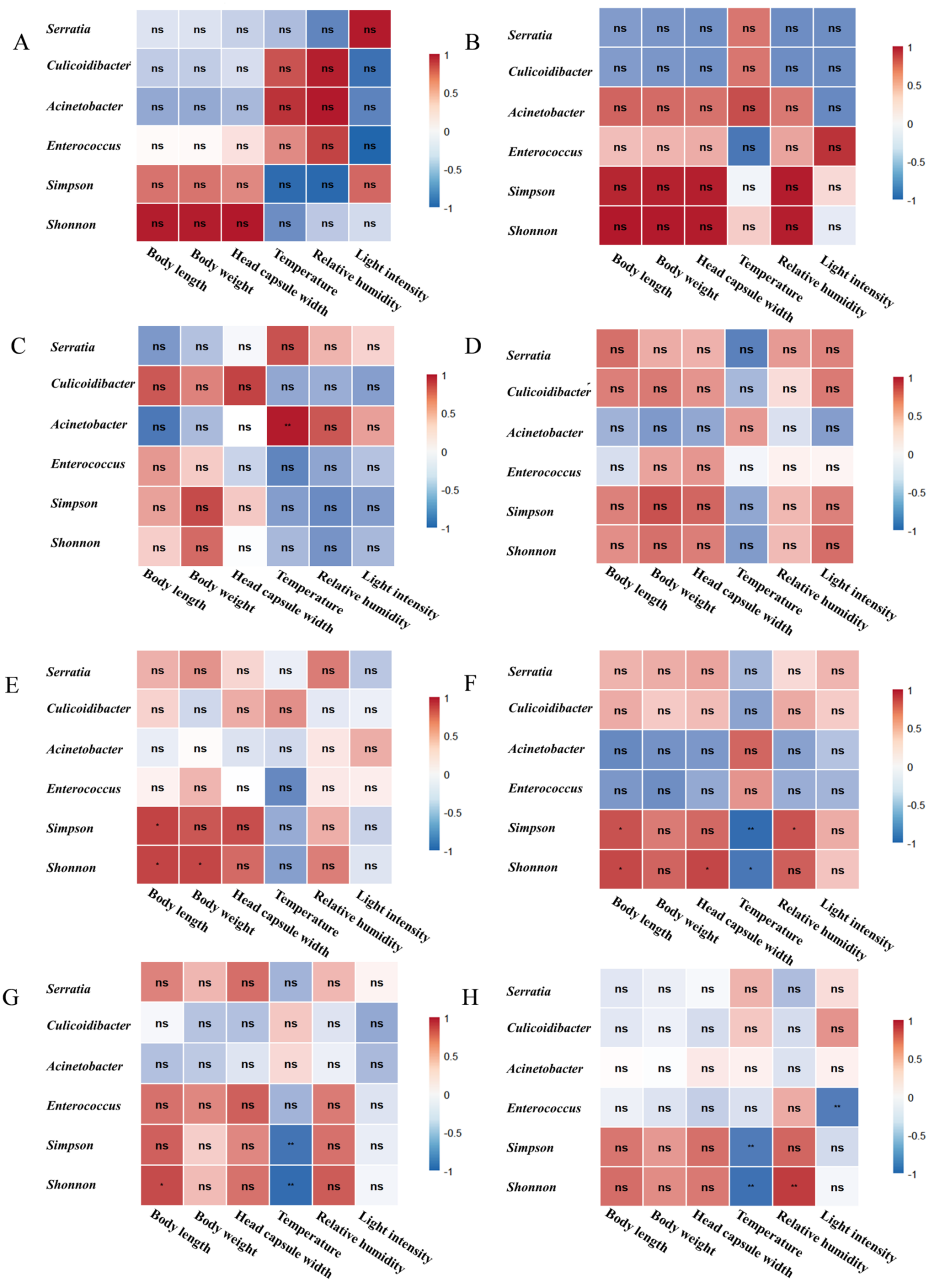
(A‐H): They are respectively for ages 1 to 8 Correlation analysis between selected strains and physical index, environmental factors (*spearman*): * *p* < 0.05, **p < 0.01.

Correlation analysis may reveal significant relationships between gut microbiota diversity, host morphological indices, and environmental factors (Figure 8A–H). Specifically, the Shannon diversity index in 5th–7th instar larvae may show a positive correlation with body length, while alpha diversity in 6th–8th instar larvae may negatively correlate with temperature. At the genus level, *Acinetobacter* abundance in 3rd instar larvae may positively associate with temperature.

## 4 Discussion

The gut microbiota played a pivotal role in insect physiology, behavior, and ecological adaptation (Jang and Kikuchi 2020). Utilizing high‐throughput 16S rRNA gene sequencing, this study characterized the gut microbiome across the larval, pupal, and adult stages of *T. issoria*. Some studies had found that the intestines of insects contain an astonishing number of microorganisms, and the abundance of the microbiota in most insects increases as they developed and matured (Caccia et al. 2019; Kim et al. 2017). Our findings revealed a dynamic successional pattern: microbial alpha‐diversity peaked in early‐instar larvae, declined during the pupal stage, and subsequently rebounded in adults. This observed trajectory aligns with the profound physiological transitions of holometabolous development. The high initial diversity in early‐instar larvae likely reflected that a diverse microbiota may enhance metabolic versatility, aiding in the digestion of varied plant compounds and improving environmental adaptability (Jones et al. 2019). As larvae developed, diversity exhibited a U‐shaped trend, decreasing before increasing again. This pattern may stem from a regulatory process in which the maturation of the larval intestinal immune system actively prunes incompatible microorganisms to form a more streamlined and functionally specialized core community, while the enhanced chewing ability and greater food intake in later instars demand a more abundant and complex microbial consortium to assist in digestion (Guo et al. 2023). The most dramatic shift occurred at the pupal stage, where diversity reached its lowest point. This bottleneck is likely a direct consequence of pre‐pupal gut clearance, the complete cessation of feeding, and the extensive histolysis and tissue reorganization characteristic of metamorphosis (Shao et al. 2024). This vulnerable, nonfeeding period represents a stringent filter for microbial persistence. Finally, the beginning of feeding by most lepidoptera adults was expected to lead to changes in microbial composition, such as the diversity and abundance had increased (Nijhout and Williams 1974).

Beta diversity analysis revealed significant compositional shifts in the gut microbiota across developmental instars of *T. issoria*. Adults and larvae had independent microbial communities, probably because the morphology and biochemistry of the digestive system changed from larvae to adults, and their diets were completely different before and after (Yang et al. 2022; Dong et al. 2020; Chen et al. 2016). Other lepidoptera insects had observed that different diets created different biochemical conditions in the intestines, thereby promoting the colonization of different microbial communities (Jordan et al. 2023).

Some specific microbial taxa could help hosts utilize life‐stage‐specific resources by providing functions related to digestion, detoxification, and nutritional supplementation (Acevedo et al. 2017; Anand et al. 2010). Consistent with patterns reported in many other Lepidoptera, the gut microbiota of *T. issoria* was consistently dominated by Pseudomonadota (formerly Proteobacteria) and Bacillota (formerly Firmicutes) across all life stages, with Bacteroidota and Actinobacteriota also constituting substantial components (Shao et al. 2024; Gohl et al. 2022; Yang et al. 2022). The persistent dominance of Pseudomonadota and Bacillota was likely functionally significant. These phyla were known to harbor genes encoding various plant cell wall‐degrading enzymes (e.g., cellulase, hemicellulase, pectinase), which were crucial for the digestion of plant material in herbivorous larvae (Li et al. 2022). In our study, a notable increase in the relative abundance of Bacillota was observed specifically during the pupal stage. Given that the pupal stage was a vulnerable, nonfeeding period characterized by developmental reorganization, an enhanced presence of Bacillota may be instrumental in supporting host defense. Members of this phylum were often involved in modulating host innate immunity and enhancing disease resistance, potentially forming a mutualistic relationship that helped protect the dormant pupa from external pathogens (Jordan et al. 2023). The Actinobacteria had the highest relative abundance during the larval stage and was renowned for its strong antibacterial properties and antibiotic resistance (Fatahi‐Bafghi 2019). Larval phase as the primary stage for nutrient intake, the presence of Actinobacteria may enhance the ability of larvae to digest food and resist defense compounds from the host plant.

At the genus level, it had been suggested that some lepidoptera species may contain a core microbiome, such as *Acinetobacter* and *Sarratia* (Proteobacteria) and *Enterococcus* and *Culicoidibacter* (Firmicutes) (González‐Serrano et al. 2020). *Acinetobacter*, widely presented in the larval stage, may be involved in metabolizing plant secondary metabolites like polyphenols and modulating insect immune responses (Mason et al. 2016; Li et al. 2021). *Serratia*, enriched in 8th‐instar larvae, may degrade aromatic compounds such as purine alkaloids (Lawrence 1998). Although often an opportunistic pathogen at high abundance, its presence at moderate levels may contribute to detoxification (Rojas‐Avelizapa et al. 2002). Through modification of the AS1 strain of *Serratia*, the researchers achieved precise targeting of multiple mosquito‐borne pathogens, demonstrating a successful proof‐of‐concept for gut microbiota‐mediated control strategies in insect vectors (Hu et al. 2025). *Serratia entomophila* was a commercially available bacterium widely used for the biocontrol of the grass grub (*Costelytra zealandica*) in pastures in New Zealand (Pritam and Sukanta 2013). Targeting or regulating *Serratia* populations at this stage could potentially suppress larval development and thereby achieve biocontrol effects. *Culicoidibacter*, widely present in mid‐ to late‐stage larvae and first isolated from *Culicoides sonorensis*, represents a genus warranting further study (Neupane et al. 2020). *Enterococcus* was the most common intestinal bacteria in Lepidoptera and *was* overwhelmingly dominant in pupae (Chen et al. 2016; Broderick et al. 2004; Shao et al. 2014; Xiang et al. 2006). Previous studies had indicated that *Enterococcus* was mainly involved in genes encoding digestive enzymes and carbohydrate metabolism, thereby affecting digestion and growth (Xia et al. 2020). Additionally, *Enterococcus* strains could produce antioxidants like catalase and superoxide dismutase, which may mitigate oxidative stress (Mazumdar et al. 2021). Recent studies revealed that *Enterococcus* could act synergistically with *Bacillus thuringiensis* (Bt) to enhance insecticidal toxicity, demonstrating that nonpathogenic functional microorganisms could be harnessed as specific biocontrol agents for precision pest management (Chen et al. 2024).

Linear discriminant analysis Effect Size (LEfSe) identified stage‐specific biomarker taxa. The *Burkholderia‐Caballeronia‐Paraburkholderia* complex was significantly enriched in 1st‐instar larvae; recent evidence suggested this group could influence host molting and development (Wang et al. 2025). *Enterobacter*, enriched in 8th‐instar larvae, may have been linked to improved larval size and fitness (Hamden et al. 2013). In adults, *Pseudomonas* and *Staphylococcus* were predominant. These genera may participate in the nutrient cycle as co‐organisms, while they were recognized as potential pathogens, particularly in immunocompromised hosts (Ma et al. 2021; Morales‐Jiménez et al. 2013; Sommer et al. 2025). Their ecological roles in the healthy adult gut, such as nutrient cycling, merit further investigation. These dynamic, stage‐specific shifts in microbial composition were likely to hold significant biological implications for host physiology across development.

To infer potential functional capacities, we employed PICRUSt_2_. The predictions indicated that the gut microbiota of *T. issoria* is extensively involved in key metabolic pathways, including amino acid metabolism, carbohydrate metabolism, energy metabolism, and metabolism of cofactors and vitamins. This suggested that nutrients derived from leaf consumption may not fully meet larval metabolic demands, necessitating complementary functions from gut microbes (Chen et al. 2020). Notably, predictive functions related to carbohydrate metabolism were significantly high, and the metabolism of amino acids, cofactors, and vitamins was significantly lower in pupae compared to other stages. This may reflect the unique physiology of the pupal stage: holometabolous insects accumulated sufficient nutrient reserves (carbohydrates stored as glycogen or trehalose) prior to metamorphosis. The observed drop in hemolymph trehalose at pupation aligns with a reported increase in carbohydrate metabolic activity (Suzuki et al. 2023). While pathways related to amino acid, cofactor, and vitamin metabolism were significantly reduced compared to other stages, we speculated that this reduction in microbial functional potential could be attributed to two biological factors. First, pupae ceased feeding during metamorphosis, resulting in a sharp decline in external nutrient input. Consequently, microbial growth was restricted, and the nutrients on which many microorganisms relied for survival were exhausted (Dittmer and Brucker 2021). Second, extensive gut tissue remodeling occurred during pupation, which involved histolysis and reconstruction of the intestinal epithelium. This process would have altered gut microenvironmental conditions, potentially restricting the colonization, abundance, and metabolic activity of gut microbiota (Zhang et al. 2018). Therefore, the observed downregulation of metabolic pathways was likely a combined consequence of halted feeding and host physiological adaptation during metamorphosis.

This study indicated that temperature may influence the diversity of insect gut microbiota. With increasing temperature, microbial diversity tended to decrease. Elevated temperatures may reduce microbial diversity, alter bacterial composition, and negatively affect insect survival and adaptability (El‐Shesheny et al. 2016; Gerber et al. 2021; Mason and Shikano 2023). Previous studies had shown that gut microbiota could help the host adapt to the stress brought by the outside world, such as dual stress from entomopathogens and herbicides (Zhang et al. 2024). Meanwhile, the gut microbiota also played an essential role in insect thermal adaptation, responding to temperature fluctuations through structural reorganization and metabolic cooperation with the host (Hafsi et al. 2024; Tang, Li, et al. 2025). The effects of temperature‐mediated shifts in gut microbiota on host physiology and their implications for biological control warranted further investigation.

In summary, this was the first description of the overall structure of gut microbiota across the life stages of *T. issoria*. In this study, we found the dynamics of microbiota diversity, community composition and potential functions across different developmental stages of *T. issoria*, enriching the intestinal microbial resources of lepidoptera insects. Several aspects of this study point to valuable directions for future research. First, while the functional predictions based on PICRUSt2 provide useful insights, they required experimental validation. And, although rarefaction was applied to minimize bias caused by uneven sequencing depth across samples, this normalization process may lead to loss of information. Future studies incorporating metagenomic sequencing, transcriptomics, or metabolomics could directly confirm the functional gene repertoire of the gut microbiota. Second, the temperature‐related observations under the laboratory conditions (25°C ± 5°C) should be extended. Future studies employing controlled gradient temperature experiments could establish direct causal relationships between temperature and gut microbial diversity, structure, and function, thereby clarifying the adaptive mechanisms of gut microbial communities under thermal stress. A more comprehensive understanding of these interactions could pave the way for the development of innovative biological control strategies, using functional bacteria to enhance the efficacy of biological control agents.

## Author Contributions

**Xin Yang:** data curation (equal), formal analysis (equal), investigation (equal), validation (equal), visualization (equal), writing – original draft (lead), writing – review and editing (lead). **Liu liu Dong:** data curation (supporting), investigation (supporting). **Xiao xiao Jin:** project administration (equal), visualization (equal). **Xu jie Liu:** methodology (equal), supervision (equal). **Min Gao:** investigation (equal), resources (equal). **Jie Fang:** funding acquisition (lead), project administration (equal), resources (equal), supervision (lead).

## Funding

This research was funded by Global Environmental Fund: Ground monitoring of ecological environment in Huangshan Scenic Area (grant number: K160138311).

## Conflicts of Interest

The authors declare no conflicts of interest.

## Supporting information


**Figure S1:** (A) Shannon–Wiener curves (B) Species accumulation curves.
**Table S1:** Alpha diversity index and number of species observed in the structure of microbiota.
**Table S2:** Results of the Kruskal–Wallis rank sum of Alpha diversity index.
**Table S3:** PERMANOVA of the bacterial communities of *T. issoria* at different stages.
**Table S4:** Abundance of KEGG functional prediction (%).
**Figure S2:** Cladogram indicates the phylogenetic distribution of microbiota communities across different life stages.

## Data Availability

The data that support the findings of this study are available in Genome Sequence Archive (GSA) at URL: https://ngdc.cncb.ac.cn/gsa/s/7B8bqVT5.
