# Supplementary material for: Composition and Diversity Characteristics of Gut Microbiota during the Development of Telchinia issoria (Lepidoptera: Nymphalidae)
Source: Ecol Evol. 2026 Apr 30;16(5):e73596. doi: 10.1002/ece3.73596 (PMC13132806; doi:10.1002/ece3.73596)
Supplement: Supplementary file 1 — Figure S1: (A) Shannon–Wiener curves (B) Species accumulation curves. Table S1: Alpha diversity index and number of species observed in the structure of microbiota. Table S2: Results of the Kruskal–Wallis rank sum of Alpha diversity index. Table S3: PERMANOVA of the bacterial communities of T. issoria at different stages. Table S4: Abundance of KEGG functional prediction (%). Figure S2: Cladogram indicates the phylogenetic distribution of microbiota communities across different life stages. [file ECE3-16-e73596-s001.pdf]

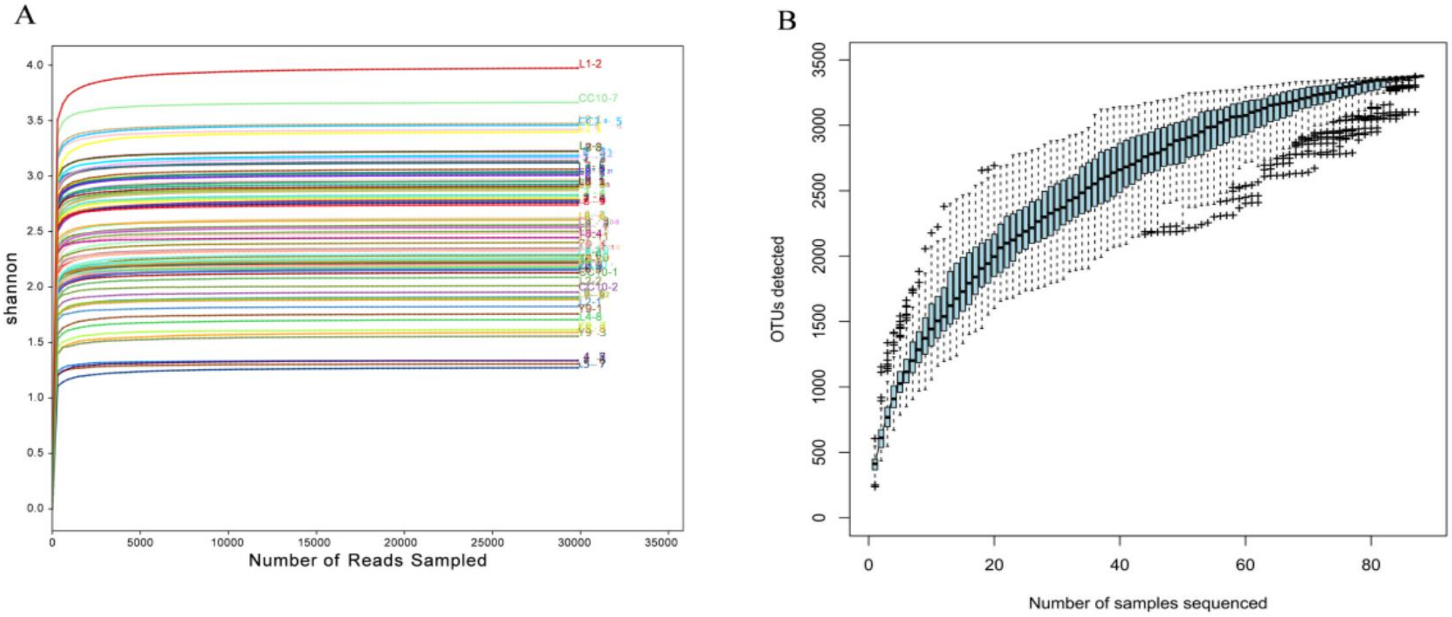

Figure S1.(A).Shannon-Wiener curves (B).species accumulation curves

Table S1.Alpha diversity index and number of species observed in the structure of microbiota.

	Observed_species	Chao1	Shannon	Simpson	Goods_coverage
"L1-1"	428	589.8	3.0854	0.8902	0.9958
"L1-2"	574	680.43	3.9612	0.8187	0.9968
"L1-3"	534	649.01	2.1241	0.9361	0.9966
"L2-1"	389	521.47	3.267	0.8988	0.996
"L2-2"	421	531.12	3.0783	0.6958	0.9967
"L2-3"	453	609.44	2.1535	0.7751	0.9956
"L3-1"	395	514.1	2.5456	0.7375	0.9958
"L3-2"	368	455.55	2.7565	0.7947	0.9967
"L3-3"	377	520.52	2.294	0.6738	0.9957
"L3-4"	391	511.12	2.6671	0.7766	0.9963
"L3-5"	338	405.16	1.9872	0.5565	0.997
"L3-6"	498	639.44	2.734	0.7097	0.9954
"L4-1"	408	496.23	2.7141	0.8043	0.996
"L4-2"	337	436	2.1016	0.7035	0.9966
"L4-3"	361	492.32	2.5535	0.8164	0.9963
"L4-4"	402	505.62	1.9523	0.6786	0.9956
"L4-5"	216	284.7	0.9712	0.3473	0.9973
"L4-6"	224	328.07	0.9435	0.3258	0.9968
"L4-7"	318	330.88	0.9465	0.272	0.9982

"L4-8"	361	374.58	1.458	0.5629	0.998
"L5-1"	469	565.4	2.6721	0.8061	0.9957
"L5-2"	467	633.54	3.1613	0.8882	0.9948
"L5-3"	429	522.94	2.4869	0.7087	0.9961
"L5-4"	445	623.36	2.3941	0.681	0.9953
"L5-5"	485	598.61	2.853	0.8496	0.9953
"L5-6"	505	658.67	2.9561	0.825	0.9951
"L5-7"	469	628.85	0.8802	0.2309	0.9931
"L5-8"	587	1079.03	1.2273	0.3318	0.9887
"L5-9"	348	442.32	1.8462	0.5763	0.9963
"L5-10"	332	433.27	2.0381	0.6949	0.9963
"L5-11"	327	395.06	2.1623	0.7318	0.9972
"L5-12"	309	414.82	2.015	0.678	0.9966
"L6-1"	347	442.02	2.9908	0.8773	0.9967
"L6-2"	374	482.9	2.9933	0.8592	0.9968
"L6-3"	297	373.56	2.0113	0.7234	0.997
"L6-4"	335	428	2.9368	0.8691	0.9969
"L6-5"	335	436.86	2.4162	0.7324	0.9969
"L6-6"	311	436.51	1.5711	0.4968	0.9959
"L6-7"	360	520.71	2.484	0.8326	0.9957
"L6-8"	262	392.63	1.5888	0.5651	0.9964
"L6-9"	409	507.61	1.8345	0.5999	0.9959
"L6-10"	418	444.22	2.6798	0.8127	0.9974
"L6-11"	418	575.2	3.2991	0.9012	0.9956
"L6-12"	523	676.79	3.201	0.9025	0.9941
"L7-1"	367	475.28	3.0106	0.8535	0.9967
"L7-2"	309	378.51	3.0527	0.8989	0.9974
"L7-3"	353	454.22	3.2514	0.9107	0.9967
"L7-4"	506	618.51	2.9736	0.8778	0.9949
"L7-5"	471	693.82	3.1573	0.911	0.9942
"L7-6"	424	550.04	2.6325	0.7819	0.9952
"L7-7"	424	547.34	3.0211	0.8911	0.9951
"L7-8"	394	557.13	2.3227	0.7012	0.9951
"L7-9"	470	621.13	3.1887	0.9039	0.9948
"L7-10"	415	584.74	2.428	0.7784	0.9947
"L7-11"	257	312.86	2.1695	0.6985	0.9977
"L8-1"	405	479.27	3.5153	0.9308	0.9967
"L8-2"	373	489.43	3.1293	0.8802	0.9965
"L8-3"	357	499.5	2.6098	0.8181	0.9961
"L8-4"	231	355.62	2.4344	0.8422	0.9973
"L8-5"	550	679.12	2.9459	0.8892	0.9945
"L8-6"	339	466.65	2.5587	0.8521	0.996
"L8-7"	295	429.19	2.1933	0.7658	0.9961
"L8-8"	381	419.07	3.1894	0.9199	0.9973

"L8-9"	349	462	2.8412	0.8718	0.9962
"L8-10"	446	626	2.5757	0.7775	0.9951
"L8-11"	425	576.68	3.183	0.9013	0.9956
"L8-12"	425	536.8	2.9055	0.8804	0.9956
"Y9-1"	326	435.78	2.0112	0.6836	0.9966
"Y9-2"	392	549.83	2.5349	0.7618	0.9959
"Y9-3"	347	522.53	2.5407	0.8238	0.9961
"Y9-4"	415	476.62	3.1767	0.8673	0.997
"Y9-5"	277	362.08	1.9364	0.6849	0.9972
"Y9-6"	313	396.07	2.4077	0.7669	0.9971
"Y9-7"	319	378.25	2.3503	0.7712	0.9973
"Y9-8"	373	443.87	2.5345	0.7605	0.9968
"CC10-1"	341	428.21	2.3756	0.7483	0.9969
"CC10-2"	250	352	1.6048	0.6426	0.9972
"CC10-3"	258	364.95	1.8915	0.7158	0.9969
"CC10-4"	361	481.68	3.6477	0.9332	0.9968
"CC10-5"	404	434.08	3.5968	0.9365	0.9977
"CC10-6"	357	452.23	2.6246	0.7871	0.9969
"CC10-7"	385	482.23	3.7092	0.9499	0.9969
"CC10-8"	341	447.31	2.3534	0.7753	0.9968

Table S2. The results of the Kruskal-Wallis rank sum of Alpha diversity index .

	**Simpson**	**Shannon**
Pupa-Larva	0.001	
Pupa-Adual	0.009	0.144
Larva-Adual	0.827	

	**Simpson**	**Shannon**
4L-5L	0.375	0.231
4L-2L	0.209	0.002
4L-3L	0.098	0.035
4L-6L	0.054	0.042
4L-7L	0.002	0.003
4L-8L	0.001	0.001
4L-1L	0.006	0.002
5L-2L	0.489	0.023
5L-3L	0.328	0.238
5L-6L	0.17	0.349
5L-7L	0.013	0.045
5L-8L	0.005	0.022
5L-1L	0.029	0.016

2L-3L	0.952	0.264
2L-6L	0.86	0.108
2L-7L	0.369	0.418
2L-8L	0.284	0.515
2L-1L	0.284	0.916
3L-6L	0.886	0.678
3L-7L	0.284	0.625
3L-8L	0.194	0.49
3L-1L	0.23	0.218
6L-7L	0.259	0.275
6L-8L	0.157	0.176
6L-1L	0.223	0.082
7L-8L	0.8	0.816
7L-1L	0.691	0.348
8L-1L	0.827	0.435

Table S3 PERMANOVA of the bacterial communities of *T.issoria*. at different stage

**Source**	**Df**	**SS**	**F**	**R^2**	**p**	**sig**
L8 vs L1	1	0.7594846	3.468597	0.21061885	0.011	.
L8 vs L5	1	1.4333139	8.095741	0.26899957	0.001	*
L8 vs L4	1	1.6487681	10.401365	0.36622764	0.001	*
L8 vs L6	1	1.2472739	7.568264	0.25595902	0.001	*
L8 vs L3	1	1.2608374	8.707147	0.3524141	0.002	*
L8 vs L7	1	0.4451686	2.313567	0.09923694	0.038	.
L8 vs L2	1	0.6087597	3.207938	0.1979239	0.012	.
L1 vs L5	1	0.7808153	4.161514	0.24249109	0.003	*
L1 vs L4	1	0.8217408	5.29236	0.37029292	0.003	*
L1 vs L6	1	0.8190685	4.907245	0.27403685	0.003	*
L1 vs L3	1	0.750924	6.104799	0.46584452	0.017	.
L1 vs L7	1	0.7754483	3.599887	0.23076366	0.006	*
L1 vs L2	1	0.4718183	1.866504	0.31816293	0.3	
L5 vs L4	1	0.3313283	2.438289	0.11930005	0.073	
L5 vs L6	1	0.1536414	1.050263	0.04556403	0.357	
L5 vs L3	1	0.4072934	3.412676	0.17579626	0.014	.
L5 vs L7	1	0.8942007	5.168185	0.1974988	0.001	*
L5 vs L2	1	0.6904798	4.35815	0.25107227	0.003	*
L4 vs L6	1	0.7387688	6.109424	0.25340397	0.001	*
L4 vs L3	1	0.3640846	5.034477	0.29554634	0.01	.
L4 vs L7	1	1.304714	8.558179	0.33485088	0.001	*
L4 vs L2	1	0.7392995	6.536641	0.42072419	0.008	.
L6 vs L3	1	0.6523097	6.363119	0.28453629	0.001	*
L6 vs L7	1	0.6666693	4.16161	0.16539521	0.001	*

L6 vs L2	1	0.6985004	5.072016	0.28065579	0.004	*
L3 vs L7	1	1.1185672	8.163515	0.35242988	0.001	*
L3 vs L2	1	0.483933	7.035036	0.50124817	0.011	.
L7 vs L2	1	0.6240929	3.395819	0.2205676	0.004	*

**Source**	**Df**	**SS**	**F**	**R2**	**P**	**sig**
pupa vs adual	1	0.8522803	6.49501	0.3169069	0.002	*
pupa vs larva	1	2.8097994	14.19413	0.1646763	0.0015	*
adual vs larva	1	2.421451	11.29505	0.1356029	0.0015	*

Table S4.Abundance of KEGG functional prediction (%)

	L1	L2	L3	L4	L5	L6	L7	Pupa	Adual
Amino acid metabolism	13.84	11.63	12.53	12.40	12.78	13.12	12.83	11.01	13.73
Biosynthesis of other secondary metabolites	1.17	1.00	1.10	1.08	1.16	1.19	1.10	0.82	1.22
Cancer: overview	0.15	0.10	0.13	0.13	0.13	0.12	0.09	0.04	0.12
Cancer: specific types	0.23	0.14	0.15	0.14	0.14	0.14	0.12	0.05	0.16
Carbohydrate metabolism	16.67	18.96	15.95	14.86	15.04	15.31	17.30	21.43	17.28
Cardiovascular disease	0.08	0.03	0.01	0.01	0.01	0.01	0.01	0.01	0.04
Cell growth and death	1.02	0.99	0.99	1.05	1.04	0.97	0.86	0.82	0.90
Cell motility	1.85	1.73	0.84	0.54	1.27	1.71	1.80	2.52	2.60
Cellular community - prokaryotes	0.16	0.17	0.19	0.19	0.18	0.19	0.18	0.18	0.21
Circulatory system	0.06	0.03	0.01	0.01	0.01	0.01	0.02	0.01	0.07
Digestive system	0.04	0.05	0.02	0.01	0.01	0.02	0.04	0.06	0.04
Drug resistance: antimicrobial	0.02	0.00	0.01	0.00	0.00	0.01	0.01	0.01	0.03
Endocrine and metabolic disease	0.16	0.18	0.20	0.21	0.20	0.20	0.18	0.18	0.14
Endocrine system	0.65	0.39	0.61	0.70	0.63	0.58	0.52	0.28	0.43
Energy metabolism	9.10	9.62	11.46	11.44	10.60	10.08	8.51	8.08	8.07
Environmental adaptation	0.23	0.25	0.23	0.25	0.27	0.28	0.29	0.25	0.27
Excretory system	0.07	0.05	0.10	0.10	0.08	0.07	0.06	0.01	0.03
Folding, sorting and degradation	2.40	2.58	2.90	3.12	3.03	2.90	2.58	2.30	2.21
Glycan biosynthesis and metabolism	1.89	2.33	2.04	1.90	1.93	1.98	2.06	2.38	2.12
Immune disease	0.10	0.14	0.14	0.13	0.13	0.12	0.12	0.16	0.10
Immune system	0.08	0.07	0.11	0.15	0.15	0.14	0.10	0.03	0.08
Infectious disease: bacterial	0.15	0.24	0.15	0.12	0.14	0.15	0.19	0.37	0.21
Infectious disease: parasitic	0.14	0.04	0.03	0.01	0.02	0.04	0.07	0.03	0.12
Lipid metabolism	4.59	3.59	3.63	3.65	3.64	3.66	4.03	3.69	4.25

Membrane transport	7.78	9.69	7.94	7.42	7.41	7.73	9.49	11.59	9.49
Metabolism of cofactors and vitamins	6.54	5.97	7.83	8.43	8.09	7.79	6.46	4.65	5.57
Metabolism of other amino acids	3.29	3.05	3.38	3.36	3.18	3.13	3.17	2.91	3.27
Metabolism of terpenoids and polyketides	2.26	1.84	2.05	2.06	2.02	1.99	1.97	1.67	2.02
Neurodegenerative disease	0.75	0.48	0.45	0.41	0.38	0.38	0.37	0.29	0.67
Nucleotide metabolism	5.83	6.65	6.73	7.46	7.40	7.11	6.91	6.53	5.68
Replication and repair	4.55	5.15	4.86	5.28	5.43	5.29	5.10	5.10	4.63
Signal transduction	3.40	3.07	2.76	2.43	2.64	3.04	3.51	3.44	4.42
Transcription	0.86	0.87	1.00	1.17	1.23	1.17	0.95	0.82	0.83
Translation	4.83	5.97	6.04	6.68	6.70	6.34	5.63	5.58	4.62
Transport and catabolism	0.56	0.43	0.41	0.38	0.34	0.34	0.40	0.40	0.52
Xenobiotics biodegradation and metabolism	4.50	2.49	3.03	2.69	2.58	2.71	2.96	2.30	3.85

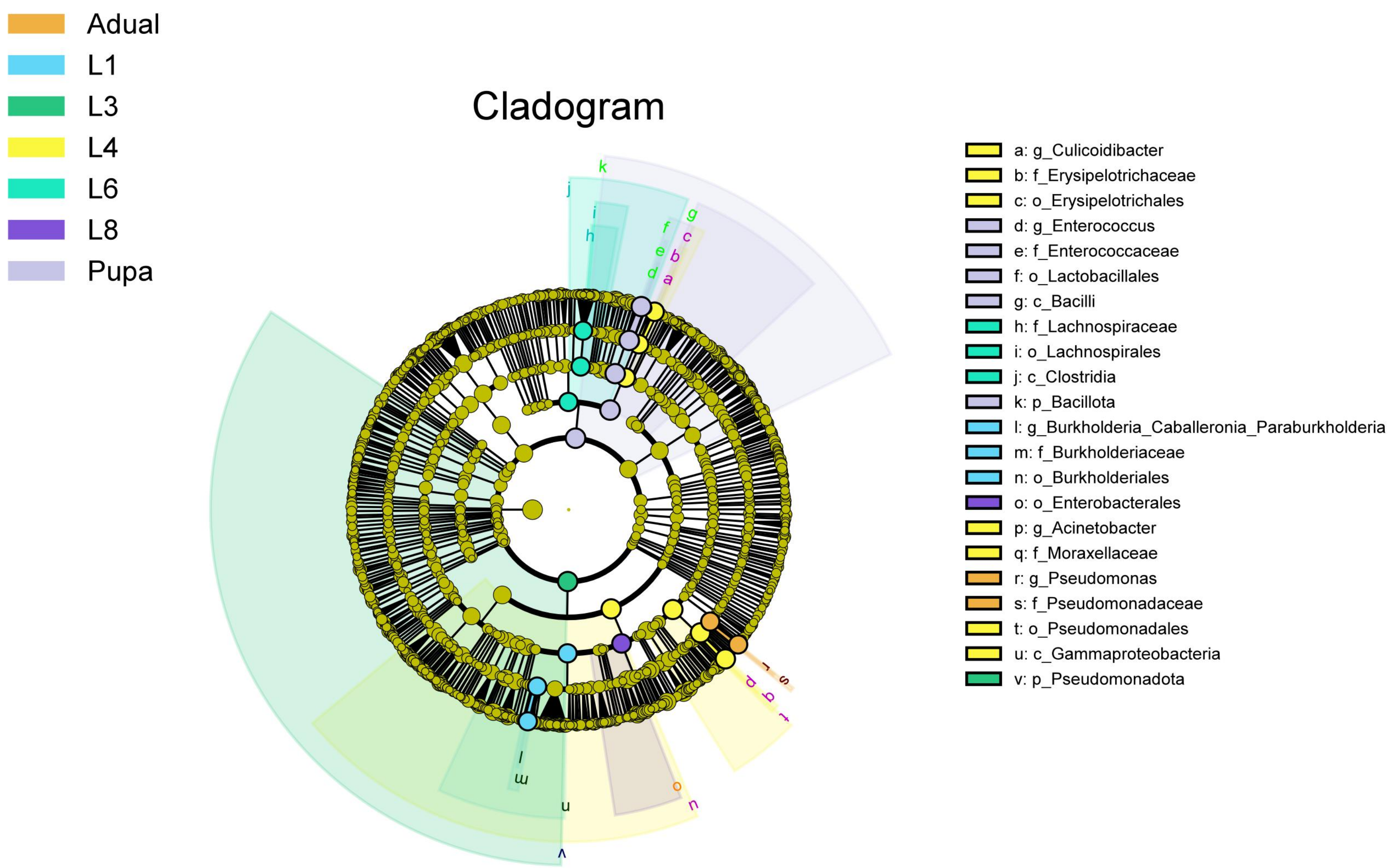
Adual
L1
L3
L4
L6
L8
Pupa
Cladogram
a: g_Culicoidibacter
b: f_Erysipelotrichaceae
c: o_Erysipelotrichales
d: g_Enterococcus
e: f_Enterococcaceae
f: o_Lactobacillales
g: c_Bacilli
h: f_Lachnospiraceae
i: o_Lachnospirales
j: c_Clostridia
k: p_Bacillota
l: g_Burkholderia_Caballeronia_Paraburkholderia
m: f_Burkholderiaceae
n: o_Burkholderiales
o: o_Enterobacterales
p: g_Acinetobacter
q: f_Moraxellaceae
r: g_Pseudomonas
s: f_Pseudomonadaceae
t: o_Pseudomonadales
u: c_Gammaproteobacteria
v: p_Pseudomonadota

Figure S2 Clado-gram indicates the phylogenetic distribution of microbiota communities across different life stages.
